# The pathway to diagnosis of type 1 diabetes in children: a questionnaire study

**DOI:** 10.1136/bmjopen-2014-006470

**Published:** 2015-03-17

**Authors:** Juliet A Usher-Smith, Matthew J Thompson, Hannah Zhu, Stephen J Sharp, Fiona M Walter

**Affiliations:** 1 The Primary Care Unit, University of Cambridge, Cambridge, UK; 2 Department of Family Medicine, University of Washington, Seattle, Washington, USA; 3 Cambridge University Hospitals NHS Foundation Trust, Cambridge, UK; 4 MRC Epidemiology Unit, University of Cambridge, Institute of Metabolic Science, Cambridge, UK

**Keywords:** PRIMARY CARE

## Abstract

**Objective:**

To explore the pathway to diagnosis of type 1 diabetes (T1D) in children.

**Design:**

Questionnaire completed by parents.

**Participants:**

Parents of children aged 1 month to 16 years diagnosed with T1D within the previous 3 months.

**Setting:**

Children and parents from 11 hospitals within the East of England.

**Results:**

88/164 (54%) invited families returned the questionnaire. Children had mean±SD age of 9.41±4.5 years. 35 (39.8%) presented with diabetic ketoacidosis at diagnosis. The most common symptoms were polydipsia (97.7%), polyuria (83.9%), tiredness (75.9%), nocturia (73.6%) and weight loss (64.4%) and all children presented with at least one of those symptoms. The time from symptom onset to diagnosis ranged from 2 to 315 days (median 25 days). Most of this was the appraisal interval from symptom onset until perceiving the need to seek medical advice. Access to healthcare was good but one in five children presenting to primary care were not diagnosed at first encounter, most commonly due to waiting for fasting blood tests or alternative diagnoses. Children diagnosed at first consultation had a shorter duration of symptoms (p=0.022) and children whose parents suspected the diagnosis were 1.3 times more likely (relative risk (RR) 1.3, 95% CI 1.02 to 1.67) to be diagnosed at first consultation.

**Conclusions:**

Children present with the known symptoms of T1D but there is considerable scope to improve the diagnostic pathway. Future interventions targeted at parents need to address the tendency of parents to find alternative explanations for symptoms and the perceived barriers to access, in addition to symptom awareness.

**Strengths and limitations of this study**

- This study uses a questionnaire developed from a previous interview study to explore the diagnostic pathway of children with newly diagnosed type 1 diabetes.
- It uses the Model of Pathway to Treatment as a framework to allow analysis of the factors acting at different stages in the pathway.
- The inclusion of a calendar with key events in the questionnaires and use of free text responses for internal validation and checking of prompted responses reduced bias but the data was necessarily collected retrospectively and so are subject to recall and framing bias.

## Introduction

Approximately 65 000 children are diagnosed with type 1 diabetes (T1D) each year and the incidence is continuing to increase at a rate of approximately 3% per year.^1^ ^2^ The most common symptoms are well described and include polyuria, polydipsia, weight loss and tiredness. At the early stages of the disease, however, these symptoms are often non-specific and distinguishing the children with T1D from the large number with similar symptoms and minor undifferentiated illness can therefore be difficult. This is reflected in studies which have shown that the mean duration of symptoms prior to diagnosis is over 2 weeks with a significant number of children experiencing delay in diagnosis or misdiagnosis^3^ and only one in five diagnosed at first encounter.^4–8^ Up to 80% of children additionally present in diabetic ketoacidosis (DKA),^9^ which has immediate life-threatening complications and is associated with poorer long-term diabetic control.^10–12^

While several studies have highlighted these difficulties in making the diagnosis and the features associated with diabetic ketoacidosis at diagnosis,^3–8^ ^13^ few have explored the period between symptom onset and diagnosis. Our recent qualitative interview study of parents and general practitioners (GPs) of children newly diagnosed with T1D suggested that the longest component in the diagnostic pathway is the time between onset of symptoms and the decision to seek medical help (known as the appraisal interval).^14^ The early symptoms are subtle, and even with some knowledge of T1D it took many parents several weeks of a complex decision-making process and often a physical trigger, such as weight loss or vomiting, to decide to consult a healthcare professional. Once the decision to seek help had been made almost all children were seen immediately and diagnoses were mostly prompt and managed appropriately. Parents continued to play a key role during the diagnostic interval however, with many having already made or suspected the diagnosis themselves, and several feeling that their GP did not take their concerns seriously.

This study builds on this earlier work by using a questionnaire developed from the interview findings to further explore the pathway to diagnosis of T1D in children. By using a structured questionnaire to survey a larger number of families we aimed to quantify the symptoms and their time course prior to diagnosis, the triggers and barriers to seeking help, the influence of parental prior knowledge of diabetes and the role of healthcare services.

## Methods

### Design

A questionnaire about the pathway from first symptom(s) to diagnosis was completed by the parent(s)/guardian(s)/step-parents (hereafter referred to as parents) of children aged 1 month to 16 years diagnosed with T1D within the previous 3 months.

### Recruitment

Children and parents were identified and recruited via the paediatric diabetes specialist nurses and research nurses at 11 hospitals within the East of England Diabetes Children and Young People's Network. Parents of all children aged 1 month to 16 years who were diagnosed with T1D within the previous 3 months at participating hospitals were eligible for inclusion unless their clinical team felt that this was not appropriate. Parents who failed to respond within 1 month were sent a reminder letter with a further copy of the questionnaire. Recruitment began at each site between February 2013 and April 2013, and continued across all sites until January 2014.

The clinical or research teams at all sites collected data on the age and gender of each child diagnosed during the study period and whether they had DKA at diagnosis. Each hospital used a slightly different definition of DKA but all included either pH <7.3 or bicarbonate <15 mmol/L (see online appendix table 1).

### The questionnaire

The questionnaire was developed from the findings of our previous qualitative study of parents and children recently diagnosed with T1D.^14^ It was first reviewed by an expert panel comprising paediatric diabetes consultants, a paediatric diabetes research nurse and primary care researchers, and then piloted with parents of four children recently diagnosed with T1D. In addition to their specific feedback, parents were asked to talk aloud while completing the questionnaire and then interviewed after completion to ensure face validity. Based on feedback from the parents, the questionnaire was revised.

The final questionnaire included five sections (see online supplementary file). The first included questions about the child's age, gender, postcode, ethnic background, family history of diabetes, any medically trained family members and the number of children in the household. Parents were also asked if they knew what the symptoms of diabetes in children were before their child was diagnosed, and if so, to give details of those symptoms they were aware of. The second section asked about the symptoms the children had experienced with yes/no responses for 14 symptoms and space to add the date they noticed the symptoms, what they thought the symptoms were due to at the time and how much they concerned them. The third section focused on help-seeking and asked where parents had looked for information, who they spoke to and then details on when and how they had sought medical advice. It also asked them to describe their main concern at their first appointment and whether they had considered diabetes. Parents were also asked in this section about factors contributing to their decision to seek medical advice sooner or later. The fourth section asked about the diagnosis, including whether it was made at their first appointment with a healthcare professional and, if not, how many subsequent consultations they had, and the investigations that were performed before diagnosis. The final section then asked parents if they felt there was anything that prolonged them finding out their child had diabetes and had further space for free text comments.

### Analysis

Data from the questionnaires were entered into a database and then double checked by a second researcher. Socioeconomic status was computed using postcode and the English indices of deprivation 2010 available online.^15^ The presence of DKA at diagnosis was obtained from hospital records rather than self-report. Walter *et al*'s Model of Pathways to Treatment^16^ ^17^ provided a theoretic model of the intervals that occur prior to a diagnosis. This model divides the pathway to diagnosis into two intervals prior to presentation to healthcare about a symptom (the appraisal interval from the onset of symptoms to perceiving a reason to discuss symptoms with a healthcare professional, and the help-seeking interval from that decision until presentation to a healthcare professional), and then the diagnostic interval from first presentation to a healthcare professional until diagnosis. The help-seeking interval was further subdivided into the behavioural interval (the time between perceiving the reason to discuss the symptoms with a healthcare professional to making the decision to seek help) and the scheduling interval (the time between making the decision to seek help and the first consultation).^18^ Intervals were calculated from responses to the questionnaire. Where dates were incomplete we applied midpoint rules to estimate the actual date.^19^ In cases where the responses in free text differed from the dates entered as numbers, the free text was assumed to be correct, and where there was uncertainty the researchers met to agree consensus.

Characteristics (age, gender, presence of DKA) were compared between children whose parents had and had not returned a questionnaire using a t test for age and χ^2^ test for gender and presence of DKA. All further analyses used only data from returned questionnaires. The frequency of the 14 symptoms was compared between those with and without DKA using a χ^2^ test. Cox regression was used to estimate the association between various factors and the hazard of diagnosis; if a factor was associated with an increased hazard (ie, HR greater than 1), this implied that that factor was associated with a shorter time to diagnosis, and vice versa. Time to diagnosis was from the date of the earliest symptom to the date of diagnosis, and the factors assessed were age, gender, family history of T1D, index of multiple deprivation, prior knowledge of symptoms of T1D, whether the parents suspected T1D, whether the diagnosis was made at the first consultation, whether the first consultation was with primary or secondary care and whether the child had DKA at diagnosis. A similar approach was used to assess factors associated with the length of the appraisal and help-seeking intervals (with the end of the interval being defined as the ‘event’ in the Cox model), but only the first six variables in the list above were considered, as the others do not relate to those time intervals. The Schoenfeld residuals test was used to assess the proportional hazards (PH) assumption for each covariate in each model. Whether parents suspected the diagnosis of T1D did not meet the PH assumption for the total diagnostic interval and so the Cox regression model was stratified by that variable. Logistic regression was used to estimate the association between the same factors and presence of DKA at diagnosis. All analyses were performed using STATA V.12.

Free text responses were grouped into similar categories and coded. Where individual free text responses contained several comments, these were each coded individually.

## Results

A total of 172 children were diagnosed with T1D in the 11 hospitals during the study period. Of those, eight families were not invited to take part in the study: five lived outside the hospital catchment area; one emigrated the week after diagnosis; and the clinical team felt it was not appropriate to include two. From the remaining 164 families invited to take part in the study, 88 (54%) completed and returned the questionnaire. There were no significant differences in the proportion presenting in DKA (p=0.27), mean age (p=0.77) or gender (p=0.77) between children of responders and non-responders.

One child was excluded from the analysis as they had no symptoms and the diagnosis was made on a random blood glucose test that the parents were doing at home on an intermittent basis as they had an older child with T1D. Children whose parents checked blood glucose at home after noticing symptoms remain in the analysis. Eighty-seven children are therefore included in the analysis that follows.

Table 1 shows the characteristics of the 87 children and families included in the study. The mean age was 9.34±4.5 years, 49 (56.3%) were male and 35 (40.2%) presented with DKA at diagnosis. The majority (90.8%) were white and as a group they were generally from less deprived areas of England, with 49.4% from the least deprived tertile of English Indices of Deprivation and only 10.3% from the most deprived.

**Table 1:** Child and family characteristics for those included in the study

Child and family characteristics	Number	Percentage
Gender		
Male	49	56.3
Female	38	43.7
Age		
0–5	26	29.9
6–10	20	23.0
11–16	41	47.1
Mean±SD	9.34±4.5	
Ethnicity		
White	79	90.8
Asian	2	2.3
Black	3	3.4
Mixed	3	3.4
Family history		
First degree relative(s) with T1D	7	8.0
First degree relative(s) with T2D	8	9.2
Second or third degree relative(s) with T1D	13	14.9
Second or third degree relative(s) with T2D	24	27.6
Indices of deprivation		
Least deprived tertile	43	49.4
Middle tertile	33	37.9
Most deprived tertile	9	10.3
Missing	2	2.3
Medically trained family member	9	10.3
DKA at diagnosis		
Yes	35	40.2
No	52	60.0

T1D, type 1 diabetes.

### Symptoms

Table 2 shows the frequency and duration of the 14 symptoms that were specifically asked about in the questionnaire. The most common symptoms were polydipsia (97.7%), polyuria (83.9%), tiredness (75.9%), nocturia (73.6%) and weight loss (64.4%). Most symptoms were present for a median of between 13 and 17 days. Faster breathing and vomiting both had much shorter median (IQR) durations of 0.5 (0–7.5) and 2.5 (1.5–5.5) days respectively than the other symptoms. Weight loss, vomiting and faster breathing were significantly more frequent in those children who presented in DKA (p=0.014, <0.0005 and 0.001, respectively). All the children had at least one of the four main symptoms (polydipsia, polyuria or nocturia, weight loss, or tiredness), 97.7% had two or more, 79.3% three or more and over half (50.6%) had all four symptoms.

**Table 2:** Frequency of symptoms among all children and those with and without diabetic ketoacidosis (DKA) and duration of individual symptoms

	Frequency of symptoms						Duration of symptoms		
	All (n=87)		DKA (n=35)		No-DKA (n=52)		Mean±SD	Median (IQR)	n
	n	Per cent	n	Per cent	n	Per cent			
Polydipsia	85	97.7	33	94.3	52	100	31.9±48	16 (8, 36)	77
Polyuria	73	83.9	27	77.1	46	88.5	29.8±53	14 (5, 26)	65
Tiredness	66	75.9	28	80.0	38	73.1	34.5±49.2	17 (10, 39)	53
Nocturia	64	73.6	28	80.0	36	69.2	31.3±52.1	15.5 (7, 28.5)	56
Weight loss	56	64.4	28	80.0*	28	53.8*	50.1±82.7	13.5 (7, 44)	42
Changes in behaviour/mood	48	55.2	17	48.6	31	59.6	34.3±40.8	15 (8, 42)	34
Change in appetite	45	51.7	18	51.4	27	51.9	30.7±48	14.5 (7, 39)	38
Abdominal pain	37	42.5	17	48.6	20	38.5	41.4±64.1	17 (7, 38)	25
Noctural enuresis	33	37.9	14	40.0	19	36.5	28.4±49.2	15 (5.5, 21.5)	28
Different smelling breath	31	35.6	14	40.0	17	32.7	17.5±28.7	6.5 (3, 17)	22
Vomiting	17	19.5	15	42.9*	2	3.8*	7.3±12.6	2.5 (1.5, 5.5)	8
Faster breathing	15	17.2	12	34.3*	3	5.8*	3.8±5.8	0.5 (0, 7.5)	8
Urinary incontinence	14	16.1	4	11.4	10	19.2	36.6±77.2	10 (3, 21)	10
Fever	12	13.8	6	17.1	6	11.5	25±35.8	8 (2, 55)	7

*p<0.05.

A small number of parents mentioned symptoms other than those listed in the questionnaire, these included constipation (9), headaches (3), thrush (3), blurred vision (2), dry skin (2) and different smelling urine (1).

### Diagnostic intervals

**Figure 1:**
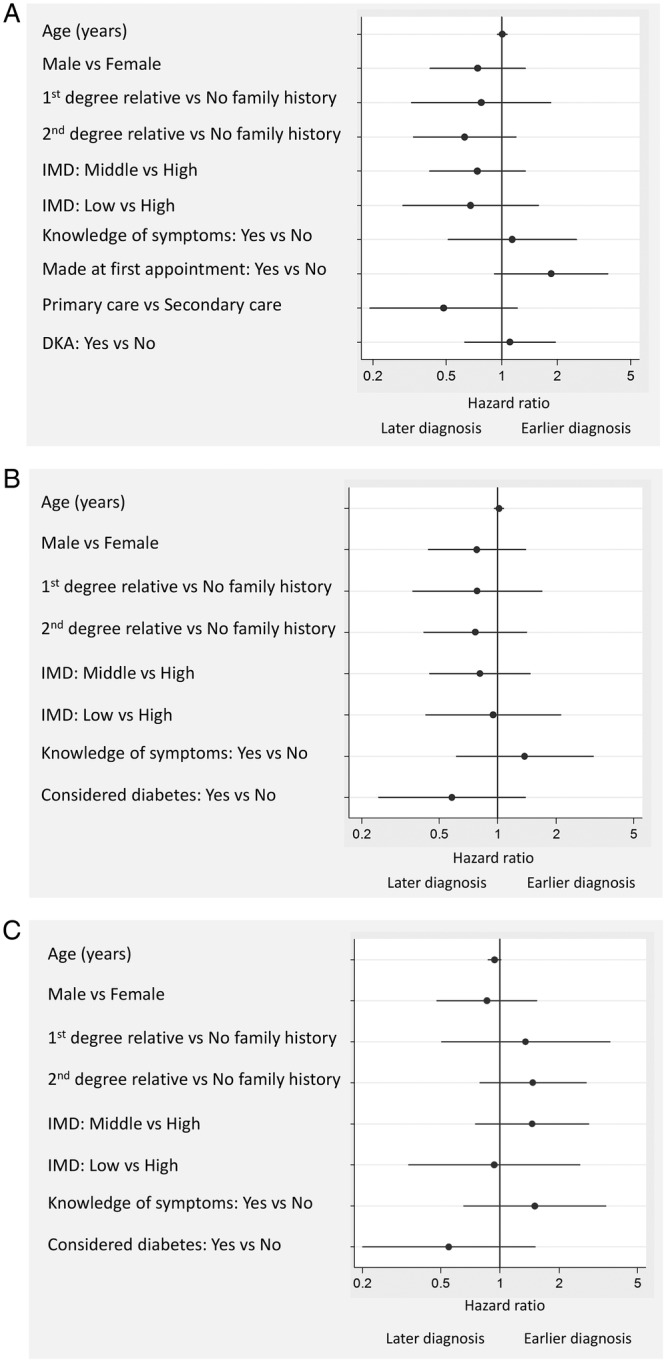
Associations between parent/child characteristics and (A) the total diagnostic interval, (B) the appraisal interval and (C) the help-seeking interval. HRs adjusted for all variables in each figure. Cox model in (A) stratified by whether parents suspected the diagnosis or not. DKA, diabetic ketoacidosis; IMD, index of multiple deprivation.

Table 3 shows the mean±SD and median (IQR) for the diagnostic intervals. Additional details on the diagnostic intervals for different subgroups are shown in online supplementary appendix table 2. The total diagnostic interval ranged from 2 to 315 days with a median (IQR) of 25 days (14–50). In unadjusted Cox regression analysis (data not shown) the time to diagnosis was significantly shorter for children diagnosed at first appointment compared with a subsequent appointment (p=0.046) and for those seen in secondary care rather than primary care (p=0.01). No evidence of associations with time to diagnosis was found for age, gender, family history of T1D, deprivation, prior knowledge of symptoms or DKA at diagnosis. In multivariable cox regression including age, gender, family history of T1D, index of multiple deprivation, prior knowledge of symptoms of T1D, whether the diagnosis was made at the first consultation, whether the first consultation was with primary or secondary care and whether the child had DKA at diagnosis (figure 1A), the association between whether the diagnosis of T1D was made at the first or subsequent appointments and total diagnostic interval remained statistically significant (p=0.022).

**Table 3:** Duration of diagnostic intervals

	Mean±SD (days)	Median (IQR) (days)	n
Appraisal interval	41±51.7	20 (9, 40)	75
Help-seeking interval	3±4.6	1 (0, 4.5)	83
Diagnostic interval	5±34.8	0 (0, 0)	83
Total diagnostic interval	48±60.4	25 (14, 50)	74

#### The appraisal interval

The appraisal interval was the longest of all the intervals in the pathway for all but three of the families with a mean±SD of 41.0±51.7 days and median (IQR) 20 (9–40) days. During this period nearly two-thirds (64%) of parents discussed the symptoms with family members, 40% with friends and 41% looked on the internet. Only 16% spoke to the child's nursery, school or playgroup and very few (6%) looked for information in books. Over half of parents (49, 56%) reported being aware of some symptoms of T1D in children prior to their child's diagnosis: 40 (45%) were aware of increased thirst, 24 (27%) of polyuria, 17 (19%) of weight loss and 13 (15%) tiredness.

Cox-regression analysis (figure 1B) showed no significant associations between parent/child characteristics and the appraisal interval.

Analysis of the free text showed that most parents found explanations for their child's symptoms (table 4). For example, polydipsia was attributed most commonly to hot weather (19/58, 33%) or infection (13/58, 22%), polyuria and nocturia were frequently explained by drinking more (29/47, 62% and 26/40, 65%) and tiredness was thought to be school related (12/44, 27%) or secondary to infection (5/44, 12%) or nocturia (4/44, 10%).

**Table 4:** Parents’ explanations for the 10 most common symptoms

Symptom	n	Number with explanation for symptom n (%)	Most common explanations n (%)	
Polydipsia	85	58 (68.2)	Hot weather	19 (32.8)
			Infection	13 (22.4)
			Activity/travel	10 (17.2)
Polyuria	73	47 (64.4)	Drinking more	29 (61.7)
			Urine infection	6 (12.7)
			Diabetes	4 (8.5)
Tiredness	66	44 (66.7)	School related	12 (27.3)
			Infection	5 (11.9)
			Nocturia	4 (9.5)
Nocturia	64	40 (62.5)	Drinking more	26 (65.0)
			Diabetes	4 (10.0)
			Urine infection	3 (7.5)
Weight loss	56	33 (58.9)	Growth related	15 (45.5)
			Decreased appetite	4 (12.1)
			Increased activity	3 (9.1)
Changes in behaviour/mood	48	31 (64.6)	Tiredness	10 (32.3)
			Age related/puberty	7 (22.6)
			Infection/illness	6 (19.4)
Change in appetite	45	28 (62.2)	Growth related	14 (50.0)
			Infection	5 (17.9)
			Holiday related	2 (7.1)
Abdominal pain	37	19 (51.4)	Infection	4 (21.1)
			School related	3 (15.8)
			Period pains	3 (15.8)
Nocturnal enuresis	33	23 (69.7)	Drinking more	13 (56.5)
			Tired	4 (17.4)
			School related	3 (13.0)
Different smelling breath	31	14 (45.2)	Poor dental hygiene	4 (28.6)
			Infection	3 (21.4)
			Diabetes	3 (21.4)

The majority of parents (61/87, 70%) additionally reported that they had suspected diabetes before their first consultation with a healthcare professional. When asked what had made them suspect diabetes, the most common reason given was that they knew the symptoms (22/59, 37%), especially thirst (12/59, 20%). Others cited information from the internet (12/59, 20%) or having a family history of diabetes (11/59, 19%).

#### The help-seeking interval

Twenty-four (28%) children were seen on the same day their parents first thought about seeking medical advice and 64 (74%) within 5 days. Most of this time was the behavioural interval (mean±SD 2.1±3.7 days, median (IQR) 0 (0–3) days) rather than the scheduling interval (mean±SD 1.1±2.6 days, median (IQR) 0 (0–1) days).

Cox-regression analysis (figure 1C) showed no significant associations between parent/child characteristics and the help-seeking interval.

The most common reasons that parents cited for seeking medical advice sooner rather than later (table 5) were that the symptoms were not getting better or were getting worse, wanting reassurance or concern something serious was wrong. This was also reflected in the free text responses where 22% of parents noted that worsening or persistent symptoms was the reason they decided to seek help. In general, fewer parents reported factors that led to them seeking medical advice later. Of those that did, the most common reason for waiting was hope that the symptoms would go away (51.6%) but 29.8% felt difficulty getting an appointment contributed and 27.6% and 25.2% were worried about wasting the GPs time or that the GP would not take them seriously, respectively.

**Table 5:** Factors influencing parents’ decisions to seek medical advice sooner or later

Factors influencing seeking medical advice	Not at all n (%)	A little n (%)	Quite a lot n (%)	Very much n (%)	Did not answer n (%)
Sooner					
Concern something serious	9 (10.3)	16 (18.4)	18 (20.7)	42 (48.3)	2 (2.3)
Symptoms getting worse	7 (8.0)	19 (21.8)	14 (16.1)	46 (52.9)	1 (1.1)
Symptoms not getting better	4 (4.6)	12 (13.8)	22 (25.3)	45 (51.7)	4 (4.6)
Wanting reassurance	8 (9.2)	15 (17.2)	16 (18.4)	46 (52.9)	2 (2.3)
Comments from family	30 (34.5)	28 (32.2)	11 (12.6)	13 (14.9)	5 (5.7)
Comments from school	63 (72.4)	10 (11.5)	4 (4.6)	4 (4.6)	6 (6.9)
Comments from friends	49 (56.3)	20 (23.0)	7 (8.0)	5 (5.7)	6 (6.9)
Written information	50 (57.5)	8 (9.2)	10 (11.5)	15 (17.2)	4 (4.6)
Later					
Difficulty getting appointment	60 (69.0)	8 (9.2)	7 (8.0)	11 (12.6)	1 (1.1)
Waiting for a particular doctor or nurse	68 (78.2)	7 (8.0)	4 (4.6)	6 (6.9)	2 (2.3)
Concern about having to wait at the surgery	72 (82.8)	6 (6.9)	4 (4.6)	3 (3.4)	2 (2.3)
Worry about wasting the doctor's or nurse's time	61 (70.1)	10 (11.5)	8 (9.2)	6 (6.9)	2 (2.3)
Worry the doctor would not take them seriously	62 (71.3)	12 (13.8)	3 (3.4)	7 (8.0)	3 (3.4)
Symptoms were not very serious	55 (63.2)	20 (23.0)	9 (10.3)	0 (0)	3 (3.4)
Hope the symptoms would go away	42 (48.3)	21 (24.1)	9 (10.3)	15 (17.2)	0 (0)
Fear of serious diagnosis	58 (66.7)	16 (18.4)	5 (5.7)	7 (8.0)	1 (1.1)

#### The diagnostic interval

**Figure 2:**
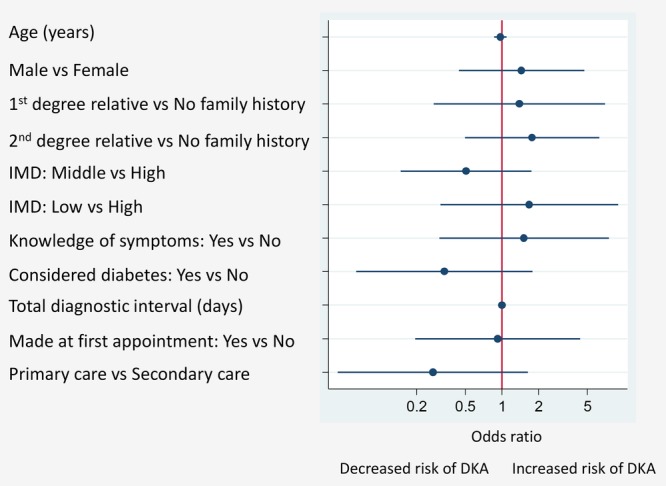
Associations between parent/child characteristics and presence/absence of DKA. ORs adjusted for all variables in the figure. DKA, diabetic ketoacidosis; IMD, index of multiple deprivation.

The diagnostic interval was the shortest of the intervals with a mean±SD of 5 days±34.8 and median 0 (IQR; 0–0) days. Sixty-nine (78%) children were diagnosed at first consultation. Cox regression was not possible given the high number of children with a diagnostic interval of zero. However, children whose parents suspected the diagnosis (n=61, 70.1%) were more likely (unadjusted relative risk (RR) 1.30, 1.02 to 1.67, p=0.046) to be diagnosed at first consultation (n=52, 85.2%) than those in whom there was no suspicion (n=26, 29.9% with 17 (65.4%) diagnosed at first consultation). All children (10) who were seen first in secondary care were diagnosed at first consultation compared with 76.6% (59/77) of those seen first in primary care, but this difference was not statistically significant (p=0.114). None of the variables considered were significantly associated with risk of DKA (figure 2).

Further details from the questionnaires were available from 14 of the 18 children who were not diagnosed at first encounter with primary care. Of these, six had fasting glucose blood tests arranged by the GP and four were given alternative diagnoses (urine infection, viral infection, tonsillitis, puberty) and diagnosed at a second appointment. Two children were diagnosed with psychological problems: In one case the child's mother had seen the GP alone to discuss her child's ‘obsessive drinking’ and was advised to see the school counsellor, and in the second the GP apparently felt the symptoms were psychological and the child was diagnosed in the emergency department four consultations later. One other family had already performed a finger prick glucose test at home which was high but the GP did not trust the result and asked the child to come back later in the day with a urine sample. In the final case, the child's mother had spoken to a health visitor and suggested diabetes but was told “no, not unless the child is lifeless”. The mother took the child to the GP 12 days later and the diagnosis was made at that consultation.

## Discussion

### Principal findings

This study shows that all children with new onset T1D present with one, and 98% present with two, of the four main symptoms of diabetes (polydipsia, polyuria, weight loss and tiredness). Moreover, over half have had symptoms for over 3 weeks before diagnosis. Most of that time is the appraisal interval during which parents found alternative explanations for the symptoms, discussed the symptoms with family and friends and looked on the internet for information. Once they made the decision to seek advice, access to healthcare was generally not difficult with 28% consulting with a healthcare professional on the same day. However, when asked about factors contributing to their decision to seek help, nearly a third of parents felt that difficulty getting an appointment contributed to them waiting to seek help and over a quarter felt that worry about wasting the doctor's time influenced their decision. This suggests that even if access is not difficult, it is perceived as such.

Once parents had sought help, one in five children were then not diagnosed at their first consultation with a healthcare professional, mainly due to being given an alternative diagnosis, most commonly infection, or waiting for further investigations. Diagnosis at first consultation was associated with a shorter total diagnostic interval and children were more likely to be diagnosed at first consultation when their parents suspected the diagnosis of T1D. The association between diagnosis at first consultation and total diagnostic interval may simply reflect the additional time between consultations, or it may be due to biological differences causing some children to develop symptoms more slowly which are then more difficult for parents as well as primary care physicians to recognise.

### Strengths and weaknesses

By using a questionnaire developed from a previous interview study^14^ and the Model of Pathway to Treatment^16^ ^17^ as a framework for analysis, this study provides in-depth insights into the diagnostic pathway of children with newly diagnosed T1D and allows factors acting at different stages in the pathway to be explored.

The main weakness is that the data were necessarily collected retrospectively and so are subject to recall and framing bias. Parents have multiple contacts with different healthcare professionals in the period immediately following diagnosis and so their responses to the questionnaire reflect a post hoc rationalisation of events framed by those subsequent encounters and increased knowledge since the diagnosis. The inclusion of a calendar with key events in the questionnaires minimised the error in recall of dates, and the free text responses allowed internal validation and checking of prompted responses. Despite these efforts, we still only have the parents’ perspective on the pathway and were not able to confirm the number of healthcare contacts, diagnostic tests or the parental reports of missed opportunities for diagnosis. We were, however, able to confirm the diagnosis of DKA from clinical records and, although there was variation in the definition of DKA used across the 11 sites, all included a biochemical measurement of either pH or bicarbonate.

Our results are also based on the views of 88 parents. Although not a large number, they were recruited from 11 sites across a large region of the UK and the response rate was over 50% with no significant differences in gender, age or DKA status between the children whose parents responded and those who did not. The fact that they were a predominantly white group from less deprived areas of England limits the generalisability of the results outside the East of England but the main findings are likely to be relevant across the UK and other countries with similar primary care healthcare provision. The questionnaire also did not include questions specifically for the children to complete and so we are unable to comment on the views of the children during this time.

### Comparison with existing literature

The median duration of symptoms prior to diagnosis was 13–17 days for the nine most frequent symptoms, with a mean of 30–50 days. This is longer than previous studies relying on retrospective review of medical records^20–23^ but similar to studies which have used a checklist to identify subtle symptoms^24^ or asked parents soon after diagnosis.^13^ ^14^ The wide range (a few days to over 6 months) has been described previously^14^ ^21^ ^23^ and highlights the heterogeneous nature of the disease.

The frequency of individual symptoms we report is also similar to previous studies.^13^ ^14^ ^20^ ^22^ ^25^ Additionally, we showed that all the children had at least one of four symptoms (polydipsia, polyuria, weight loss and fatigue) and over half (50.6%) had all four. Consistent with the known course of the disease and previous studies, vomiting,^4^ ^22^ ^24^ weight loss^13^ ^25^ ^26^ and dyspnoea^22^ were more common in those children who presented with DKA.

This is the first quantitative study to compare the time periods during the pathway to diagnosis of T1D in children. The finding that most of the total diagnostic interval was the appraisal interval is consistent with a previous qualitative study^14^ and the free text analysis confirms that during that time the parents find alternative explanations for the symptoms initially and make use of a social network of extended family, friends and work colleagues, or the internet.^14^ ^27^ ^28^ The findings that children were more likely to be diagnosed at their first encounter with a healthcare professional when their parents suspected diabetes prior to that consultation may also reflect the findings of previous qualitative work in which a number of parents prompted the GP to consider T1D and pushed for investigations.^14^ However, while parental suspicion of T1D has also been shown to be associated with a reduced risk of DKA in a parental survey,^13^ in that study the incidence of DKA at presentation was no different whether or not the parents discussed their concerns with the healthcare professional, suggesting other factors may be contributing. The absence of an effect of parental prior knowledge of diabetes either on the total diagnostic interval or the risk of DKA further highlights the complexities around the role of knowledge on help-seeking behaviour.

The finding that parents worry about wasting the doctor's time has also been shown in previous qualitative studies in children^29^ ^30^ and in studies of help-seeking behaviour for adults with symptoms of cancer in the UK^31^ ^32^ and so it may reflect a particular British trait rather than be specific to T1D or children.

### Implications for clinicians and policymakers

Clinicians should remain alert to the possibility of T1D in all children presenting with one or more symptoms of polyuria, polydipsia, weight loss and tiredness—as almost all children have at least two of these. Interventions targeted at increasing public awareness, such as the 4 T's campaign launched by Diabetes UK to raise awareness of the four most common symptoms of T1D (Toilet, Thirsty, Tired and Thinner),^33^ should continue to focus on these established symptoms.

As most of the time between symptom onset and diagnosis is the appraisal interval, the greatest benefit is likely to be seen from interventions directed towards parents and their social network, probably via the internet. Despite ongoing government pressure for better access to primary care, improving access is unlikely to have much impact on the pathway. Instead efforts should be made to address the perception that access is difficult and the general concern in the UK about wasting healthcare professional time, particularly for children with acute or sub-acute health concerns.

Additionally, although the diagnostic interval itself was generally short, one in five children presenting to primary care were not diagnosed at first consultation. Similar numbers have been reported in a recent survey in the UK which found that 24% were not diagnosed at first contact with a healthcare professional,^13^ and studies in the USA, Canada and Poland noted between 14% and 35% of children had more than one consultation before diagnosis.^6^ ^7^ ^34–36^ As in those studies, the most common reasons for not being diagnosed at first encounter was either being given an alternative diagnosis, most commonly infection, or waiting for further investigations. In this study 33% of those not diagnosed at first consultation were waiting for fasting glucose tests and in other studies the number waiting for further investigations is as high as 46%.^6^ ^13^ This suggests that healthcare professionals may have considered a diagnosis of T1D but either lack ready access to rapid tests to confirm or exclude the diagnosis, or are reluctant to use existing tests in children.^14^ Access to point of care urine and finger-prick testing and the use of those tests should be routine management for all children presenting with one or more of the four main symptoms of diabetes. The increased use of point of care testing in emergency departments may also explain why all children seen in secondary care were diagnosed at their first consultation. While educational interventions aimed at primary care physicians may help a small number of children not currently diagnosed at first encounter, finding ways to overcome barriers to point-of-care tests in primary care may be more effective and this approach may also improve the diagnosis of other serious illnesses in children and adults.

### Unanswered questions and future research

While this study contributes to our understanding of the pathway to diagnosis and the stages at which this may be improved, the findings are unable to explain the large variability in the overall duration of the pathway to diagnosis and why some children develop DKA within a few weeks while others can be symptomatic for up to 6 months before requiring treatment. Further studies are, therefore, needed into the natural course and biology of the disease to better understand these variations. The findings also highlight the need for continuing research into the presentation of serious but rare conditions in primary care and the best ways to improve diagnosis of these conditions.
